# New approach for estimating risk of miscarriage after chorionic villus sampling

**DOI:** 10.1002/uog.22041

**Published:** 2020-10-17

**Authors:** M. M. Gil, F. S. Molina, M. Rodríguez‐Fernández, J. L. Delgado, M. P. Carrillo, J. Jani, W. Plasencia, V. Stratieva, N. Maíz, P. Carretero, A. Lismonde, P. Chaveeva, J. Burgos, B. Santacruz, J. Zamora, C. De Paco Matallana

**Affiliations:** ^1^ Department of Obstetrics and Gynecology Hospital Universitario de Torrejón, Torrejón de Ardoz Madrid Spain; ^2^ School of Health Sciences Universidad Francisco de Vitoria, Pozuelo de Alarcón Madrid Spain; ^3^ Department of Obstetrics and Gynecology Hospital Clínico San Cecilio, Instituto de Investigación Biosanitaria IBS Granada Spain; ^4^ Department of Obstetrics and Gynecology Hospital Clínico Universitario ‘Virgen de la Arrixaca’, El Palmar Murcia Spain; ^5^ Institute for Biomedical Research of Murcia, IMIB‐Arrixaca, El Palmar Murcia Spain; ^6^ Department of Obstetrics and Gynecology Hospital Universitario ‘Virgen de las Nieves’ Granada Spain; ^7^ Department of Obstetrics and Gynecology, University Hospital Brugmann Université Libre de Bruxelles Brussels Belgium; ^8^ Hospiten Group Tenerife Canary Islands Spain; ^9^ Obs/Gyn Dr Shterev Hospital Sofia Bulgaria; ^10^ OSCAR Clinic Sofia Bulgaria; ^11^ Fetal Medicine Unit, Department of Obstetrics and Gynecology BioCruces Health Research Institute, Hospital Universitario Cruces, Universidad del País Vasco (UPV/EHU), Barakaldo País Vasco Spain; ^12^ CIBER Epidemiology and Public Health Clinical Biostatistics Unit, Hospital Ramón y Cajal Madrid Spain; ^13^ Barts Research Centre for Women's Health, WHO Collaborating Centre Queen Mary University of London London UK

**Keywords:** adverse pregnancy outcome, chorionic villus sampling, first‐trimester screening, invasive procedures, invasive testing, miscarriage, pregnancy complications, prenatal diagnosis

## Abstract

**Objective:**

To estimate the risk of miscarriage associated with chorionic villus sampling (CVS).

**Methods:**

This was a retrospective cohort study of women attending for routine ultrasound examination at 11 + 0 to 13 + 6 weeks' gestation at one of eight fetal‐medicine units in Spain, Belgium and Bulgaria, between July 2007 and June 2018. Two populations were included: (1) all singleton pregnancies undergoing first‐trimester assessment at Hospital Clínico Universitario Virgen de la Arrixaca in Murcia, Spain, that did not have CVS (non‐CVS group); and (2) all singleton pregnancies that underwent CVS following first‐trimester assessment at one of the eight participating centers (CVS group). We excluded pregnancies diagnosed with genetic anomalies or major fetal defects before or after birth, those that resulted in termination and those that underwent amniocentesis later in pregnancy. We used propensity score (PS) matching analysis to estimate the association between CVS and miscarriage. We compared the risk of miscarriage of the CVS and non‐CVS groups after PS matching (1:1 ratio). This procedure creates two comparable groups balancing the maternal and pregnancy characteristics that are associated with CVS, in a similar way to that in which randomization operates in a randomized clinical trial.

**Results:**

The study population consisted of 22 250 pregnancies in the non‐CVS group and 3613 in the CVS group. The incidence of miscarriage in the CVS group (2.1%; 77/3613) was significantly higher than that in the non‐CVS group (0.9% (207/22 250); *P* < 0.0001). The PS algorithm matched 2122 CVS with 2122 non‐CVS cases, of which 40 (1.9%) and 55 (2.6%) pregnancies in the CVS and non‐CVS groups, respectively, resulted in a miscarriage (odds ratio (OR), 0.72 (95% CI, 0.48–1.10); *P* = 0.146). We found a significant interaction between the risk of miscarriage following CVS and the risk of aneuploidy, suggesting that the effect of CVS on the risk of miscarriage differs depending on background characteristics. Specifically, when the risk of aneuploidy is low, the risk of miscarriage after CVS increases (OR, 2.87 (95% CI, 1.13–7.30)) and when the aneuploidy risk is high, the risk of miscarriage after CVS is paradoxically reduced (OR, 0.47 (95% CI, 0.28–0.76)), presumably owing to prenatal diagnosis and termination of pregnancies with major aneuploidies that would otherwise have resulted in spontaneous miscarriage. For example, in a patient in whom the risk of aneuploidy is 1 in 1000 (0.1%), the risk of miscarriage after CVS will increase to 0.3% (0.2 percentage points higher).

**Conclusions:**

The risk of miscarriage in women undergoing CVS is about 1% higher than that in women who do not have CVS, although this excess risk is not solely attributed to the invasive procedure but, to some extent, to the demographic and pregnancy characteristics of the patients. After accounting for these risk factors and confining the analysis to low‐risk pregnancies, CVS seems to increase the risk of miscarriage by about three times above the patient's background risk. Although this is a substantial increase in relative terms, in pregnancies without risk factors for miscarriage, the risk of miscarriage after CVS remains low and similar to, or slightly higher than, that in the general population. Copyright © 2020 ISUOG. Published by John Wiley & Sons Ltd.

## Abstract 2

This article's abstract has been translated into Spanish and Chinese. Follow the links from the abstract to view the translations.

## RESUMEN

**Nuevo enfoque para estimar el riesgo de aborto después de una biopsia de vellosidades coriónicas**

**Objetivo:**

Estimar el riesgo de aborto asociado con la biopsia de vellosidades coriónicas (BVC).

**Métodos:**

Se trata de un estudio retrospectivo de cohorte de mujeres que acudieron a un examen ecográfico de rutina entre las 11+0 y las 13+6 semanas de gestación a una de entre un total de ocho centros de medicina fetal en España, Bélgica y Bulgaria, entre julio de 2007 y junio de 2018. En el estudio se incluyeron dos poblaciones: 1) todos los embarazos con feto único sometidos a evaluación del primer trimestre en el Hospital Clínico Universitario Virgen de la Arrixaca de Murcia (España), a las que no se les hizo una BVC (grupo no BVC); y 2) todos los embarazos con feto único sometidos a BVC tras la evaluación del primer trimestre en uno de los ocho centros participantes (grupo BVC). Se excluyeron los embarazos diagnosticados con anomalías genéticas o defectos fetales importantes antes o después del nacimiento, los que resultaron en una interrupción y los que más tarde se sometieron a amniocentesis durante el embarazo. Para estimar la relación entre la BVC y el aborto espontáneo se utilizó el pareamiento por puntaje de propensión (PPP). Se comparó el riesgo de aborto de los grupos BVC y no BVC después del pareamiento PPP (razón 1:1). Este procedimiento creó dos grupos comparables en los que las características de la madre y el embarazo que se asocian con la BVC estaban equilibradas, de manera similar a cómo funciona la aleatorización en un ensayo clínico aleatorizado.

**Resultados:**

La población de estudio consistió en 22.250 embarazos en el grupo no BVC y 3.613 en el grupo BVC. La incidencia de abortos en el grupo BVC (2,1%; 77/3.613) fue significativamente mayor que en el grupo no BVC (0,9% (207/22.250); P<0,0001). El algoritmo del PPP emparejó 2.122 BVC con 2.122 casos no BVC, de los cuales 40 (1,9%) y 55 (2,6%) embarazos en los grupos BVC y no BVC, respectivamente, resultaron en un aborto espontáneo (razón de momios (RM), 0,72 (IC 95%, 0,48–1,10); P=0,146). Se encontró una interacción significativa entre el riesgo de aborto espontáneo después de una BVC y el riesgo de aneuploidía, lo que sugiere que el efecto de la BVC en el riesgo de aborto espontáneo difiere según las características del contexto. Concretamente, cuando el riesgo de aneuploidía es bajo, el riesgo de aborto después de una BVC aumenta (RM, 2,87 (IC 95%, 1,13–7,30)) y cuando el riesgo de aneuploidía es alto, paradójicamente el riesgo de aborto después de una BVC se reduce (RM, 0,47 (IC 95%, 0,28–0,76)), presumiblemente debido al diagnóstico prenatal y a la interrupción de embarazos con aneuploidías importantes que, de otro modo, hubieran provocado un aborto espontáneo. Por ejemplo, en una paciente para quien el riesgo de aneuploidía es de 1 entre 1000 (0,1%), el riesgo de aborto después de la BVC aumenta al 0,3% (0,2 puntos porcentuales más alto).

**Conclusiones:**

El riesgo de aborto espontáneo en las mujeres que se someten a una BVC es aproximadamente un 1% mayor que el de las mujeres a las que no se les hace, aunque este exceso de riesgo no se atribuye únicamente al procedimiento agresivo sino, en cierta medida, a las características demográficas y del embarazo de cada paciente. Después de tener en cuenta estos factores de riesgo y limitar el análisis a los embarazos de bajo riesgo, la BVC parece triplicar aproximadamente el riesgo de aborto en comparación con el riesgo de fondo de la paciente. Aunque se trata de un aumento sustancial en términos relativos, en los embarazos sin factores de riesgo de aborto, después de una BVC el riesgo de aborto sigue siendo bajo y similar, o ligeramente superior, al de la población en general. Copyright © 2020 ISUOG. Published by John Wiley & Sons Ltd.

## 摘要

**评估绒膜绒毛取样后流产风险的新方法**

**目的:**

评估与绒膜绒毛取样(CVS)相关的流产风险。

**方法:**

这是一项回顾性队列研究，参与对象是2007年7月至2018年6月之间在西班牙、比利时和保加利亚的八所胎儿医学中心之一参加常规超声检查的孕妇(孕期在11周至13周零6天之间)。研究包含两类群体:(1)在西班牙穆尔西亚自治区阿瑞夏卡贞女大学临床医院进行评估的处于早期妊娠的所有单胎孕妇，她们没有经历过CVS(即非CVS组)；以及(2)在八所参与研究的中心之一进行早期妊娠评估后经历过CVS的

所有单胎孕妇(即CVS组)。我们排除了在出生前后被诊断为遗传异常或重大胎儿缺陷的妊娠，即那些导致妊娠终止或在孕期晚期经历过羊膜穿刺术的孕妇。我们采用了倾向度(PS)匹配分析来评估CVS与流产之间的关联。在PS匹配(1:1的比率)后，我们对比了CVS组和非CVS组的流产风险。该过程创造出两个可比较组，平衡了与CVS相关的产妇及孕期特征，类似于随机临床试验中进行的随机化。

**结果:**

研究人群由非CVS组中的22250个妊娠和CVS组中的3613个妊娠组成。CVS组中的流产发生率(2.1%; 77/3613)比非CVS组中的流产发生率(0.9% (207/22 250); P<0.0001)要高得多。PS算法将2122个CVS与2122个非CVS相匹配，CVS组和非CVS组分别有40(1.9%)和 55(2.6%)个妊娠发生了 流产(比值比(OR)，0.72(95% CI, 0.48–1.10)；P=0.146)。我们发现，继CVS后的流产风险与异倍体风险之间有较大的相互影响，暗示了根据背景特征CVS对流产风险的影响有所不同。具体来说，当异倍体风险低时，CVS后的流产风险增加(比值比，2.87(95% CI, 1.13–7.30))，而当异倍体风险高时，CVS后的流产风险却反常地降低了(比值比，0.47(95% CI, 0.28–0.76))，这可能归因于对重大异倍体(此情况原本就会导致自发性流产)

的产前诊断和终止妊娠。例如，一个患者的异倍体风险为千分之一(0.1%)，CVS后的流产风险将增至0.3%(增加了0.2个百分点)。

**结论:**

经历过CVS的孕妇的流产风险比没有CVS的孕妇约高1%，尽管该增余风险并不单独归因于入侵性检查，但是在一定程度上，归因于患者的特定年龄段和妊娠特征。在解释了这些风险因素并将分析限制在低风险妊娠之后，CVS似乎将流产风险增加到患者背景风险的约三倍以上。尽管这相对来说是一个重大的增加，在无流产风险因素的妊娠中，CVS后的流产风险仍然是低的，类似于或略高于一般人群的情况。版权 © 2020 ISUOG。由威利父子公司(John Wiley & Sons Ltd)出版。

**CONTRIBUTION**

*What are the novel findings of this work?*

The risk of miscarriage following chorionic villus sampling (CVS) is highly dependent on the patient‐specific background risk of miscarriage. Because risk factors associated with CVS (risk factors for aneuploidy) are also associated with spontaneous miscarriage, in women at low risk for aneuploidy, CVS is associated with a significant increase in the miscarriage rate while, paradoxically, in women with high risk of aneuploidy, the risk of miscarriage after CVS is reduced, presumably owing to prenatal diagnosis and termination of pregnancies with major aneuploidies that would otherwise have resulted in spontaneous miscarriage.

*What are the clinical implications of this work?*

The true procedure‐related risk of miscarriage after CVS can only be estimated by examining women at low risk for aneuploidy. In such women, the risk of miscarriage after CVS increases by about three times. Although this is a substantial increase in relative terms, in pregnancies without prior risk factors the risk of miscarriage after CVS remains low and similar to, or slightly higher than, that in the general population.

## INTRODUCTION

Chorionic villus sampling (CVS), which was first described in 1975 and introduced into widespread clinical practice in the 1980s^1^, is a useful invasive test for the early prenatal diagnosis of chromosomal and genetic abnormalities. The procedure‐related risk of miscarriage was not investigated in studies that randomized women to CVS *vs* non‐invasive testing, but it was derived indirectly through, first, randomized controlled trials (RCTs) comparing CVS with first‐ or second‐trimester amniocentesis, and second, comparison of the rate of miscarriage in groups with similar risk factors that had CVS *vs* those that did not have invasive testing. The results of such trials established that, first, the risk of miscarriage following CVS is lower than that following early amniocentesis but similar to that following mid‐trimester amniocentesis, and second, the risk of miscarriage is similar following transabdominal and transcervical CVS^2^, ^3^, ^4^, ^5^, ^6^, ^7^. Consequently, since the only trial comparing mid‐trimester amniocentesis with expectant management reported a 1% higher risk of miscarriage in the amniocentesis group^8^, it was assumed that the risk of miscarriage following CVS was also about 1%.

Another approach for estimating the procedure‐related risk of miscarriage from CVS is to compare the rate of miscarriage between women who had CVS and those who did not have invasive testing. However, such an approach is likely to entail bias against CVS because several of the factors that lead to the need for CVS are also risk factors for miscarriage, such as increased maternal age, increased fetal nuchal translucency (NT) thickness, low serum pregnancy‐associated plasma protein‐A (PAPP‐A) and abnormal flow in the fetal ductus venosus^9^, ^10^, ^11^, ^12^, ^13^. One possible approach to overcome this problem is to use logistic regression analysis to identify predictors of miscarriage in women who did not undergo CVS and then apply the model to women who had CVS and compare the observed to the expected number of miscarriages in the latter group^13^, ^14^, ^15^. A second approach is to perform a propensity score (PS) analysis to create two homogeneous groups suitable for comparison^16^. PS analysis has emerged as a robust methodology well suited for estimating causal effects from observational data while accounting for a greater number of confounder effects than for which classical multivariate analysis could adjust^17^, ^18^. Studies utilizing these approaches have reported that the procedure‐related risk of miscarriage following CVS may be considerably lower than 1%^13^, ^14^, ^15^, ^16^. A recent meta‐analysis of seven studies reporting pregnancy outcome following CVS compared 13 011 women who underwent CVS with 232 680 women who did not have the procedure, and estimated the risk of miscarriage following CVS to be 0.20% (95% CI, −0.13 to 0.52%)^19^. However, the results of the included studies were heterogeneous and, in such cases, the value of pooled estimates from meta‐analyses is questionable^20^.

The main objective of this multicenter study was to estimate the CVS‐related risk of miscarriage after accounting for the effects of maternal and pregnancy characteristics that could have driven the decision to undergo CVS.

## METHODS

### Study design and population

This was a retrospective cohort study of women attending for routine ultrasound examination at 11 + 0 to 13 + 6 weeks' gestation at one of eight participating fetal medicine units in Spain (Hospital Clínico Universitario Virgen de la Arrixaca, Murcia, Hospital Clínico Universitario San Cecilio and Hospital Universtario Virgen de las Nieves, Granada, Hospiten de Tenerife, Tenerife and Hospital Universitario de Cruces, Bilbao), Belgium (Brugmann University Hospital, Brussels) and Bulgaria (Shterev Hospital and OSCAR Clinic, Sofia), between July 2007 and June 2018. During this visit, patient characteristics and medical history were recorded and an ultrasound examination was carried out to assess viability, diagnose major defects, measure fetal crown–rump length (CRL) and fetal NT thickness, and assess ductus venosus flow (positive or negative/reversed a‐wave). A blood sample was collected in the same visit (*n* = 651 (2.5%)) or 1–2 weeks earlier (*n* = 25 212 (97.5%)) for measurement of serum free β‐human chorionic gonadotropin (β‐hCG) and PAPP‐A. Screening for trisomies 21, 18 and 13 was carried out using The Fetal Medicine Foundation algorithm, which combines maternal age, fetal NT, ductus venosus flow and multiples of the median (MoM) values of free β‐hCG and PAPP‐A^21^. The estimated risk for trisomies was then used to counsel the women. In all patients who chose to undergo invasive testing, CVS was performed using the same transabdominal technique by or under the supervision of a fetal medicine expert trained at King's College Hospital, London, UK. Pregnancies were dated according to fetal CRL at the time of screening if they were naturally conceived^22^ and according to conception date if they were conceived by *in‐vitro* fertilization.

The following patient characteristics were recorded: maternal age, weight, height, ethnicity (white, black, South Asian, East Asian and mixed), method of conception (natural or assisted conception requiring the use of ovulation drugs or *in‐vitro* fertilization), cigarette smoking during pregnancy (yes or no), parity (parous or nulliparous if no previous pregnancy reached at least 24 weeks' gestation), and medical history of diabetes mellitus and chronic hypertension (yes or no).

Two populations were included in the study: (1) all singleton pregnancies undergoing first‐trimester assessment at Hospital Clínico Universitario Virgen de la Arrixaca in Murcia (Spain) that did not undergo CVS (control group); and (2) all singleton pregnancies that underwent CVS following first‐trimester assessment at any of the participating centers. The main indication for CVS was increased risk for aneuploidy, but other reasons were increased NT thickness, history of genetic disease in the family, previous aneuploidy or maternal request. The eligibility criteria were singleton pregnancy with a live fetus at 11 + 0 to 13 + 6 weeks without genetic anomalies or major fetal defects (such as acrania, holoprosencephaly, megacystis, exomphalos, congenital heart defects) diagnosed before or after birth. We excluded pregnancies resulting in termination for any reason, pregnancies lost to follow‐up and those that underwent amniocentesis later in pregnancy.

The primary outcome measure was the incidence of miscarriage, defined as pregnancy loss occurring before 24 weeks' gestation regardless of the interval between CVS and fetal demise. The findings of the investigations and pregnancy outcome were recorded in computer databases. Approval for the study and waiver of consent was obtained from the relevant research ethics committee of each participating center.

### Statistical analysis

Descriptive data are expressed as median and interquartile range (IQR) and as proportions (absolute and relative frequencies). Comparisons between the treatment groups were performed using the Mann–Whitney *U*‐test or two‐tailed χ^2^ test, as appropriate. Analysis was performed on a complete case basis, and the number of pregnancies included in each analysis were reported whenever necessary; the level of significance was set at 0.05.

Because we noted important differences in baseline clinical characteristics between the CVS and the non‐CVS groups, we performed a PS matching analysis to assess the effect of CVS on the risk of miscarriage, adjusting for the confounding bias caused by this imbalance. Compared with classic multivariate adjustments, the PS permits finer adjustments for wider sets of covariates. PS was defined as the conditional probability of having CVS given the measured covariates in order to balance covariates in the two groups. To obtain the PS, we fitted a logistic regression model with CVS as the dependent variable and then we modeled the conditional probability of undergoing CVS as a function of baseline and those clinical characteristics associated with having CVS. We used the PS to match, without replacement, each complete CVS case with the non‐CVS case that had the closest PS in a 1:1 ratio, in order to optimize the precision of the estimate of association and limit bias. We also accepted cases only if the difference in PS between matched cases was small (caliper of 0.1), resulting in excellent balance between the CVS and non‐CVS cases as matched samples^23^. We computed standardized differences for all variables included in the PS before and after matching, to assess the effect of matching on the imbalance, and deemed a 10% standardized difference to be the limit for a correct balance. After matching, we compared the miscarriage rate between the CVS and non‐CVS cases as matched groups. Finally, we calculated the odds ratio (OR) to quantify the association between CVS and miscarriage using univariate logistic regression analysis fitted by generalized estimating equations to account for matched data.

The statistical software package R was used for data analysis (R Foundation for Statistical Computing, Vienna, Austria)^24^. The R package MatchIt was used for matching with the PS and analysis of matched cases was performed using the R package Geepack^25^, ^26^.

## RESULTS

### Study population

**Figure 1:**
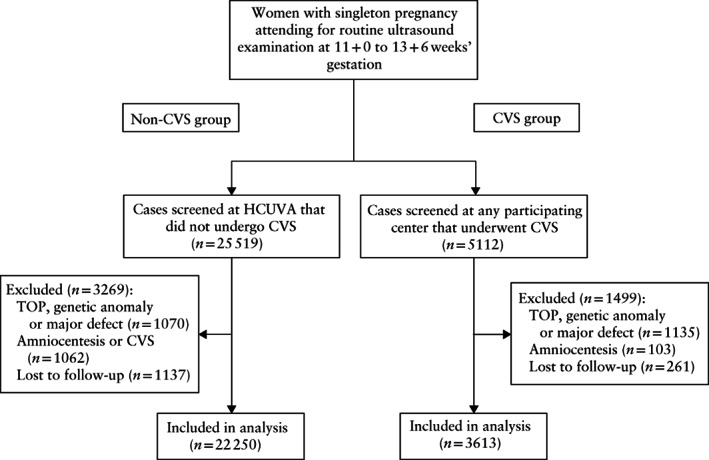
Flowchart of patients included in study. CVS, chorionic villus sampling; HCUVA, Hospital Clínico Universitario Virgen de la Arrixaca, Murcia, Spain; TOP, termination of pregnancy.

The study population consisted of 22 250 singleton pregnancies in the non‐CVS group and 3613 in the CVS group (Figure 1). In the non‐CVS group, there were 21 873 (98.3%) pregnancies with a low‐risk for aneuploidy based on the first‐trimester combined test, 345 (1.6%) with a high risk and 32 (0.1%) who declined risk assessment. Maternal and pregnancy characteristics are shown in Table 1. In the CVS group, median maternal age, fetal NT and serum free β‐hCG MoM were significantly higher and median maternal weight and PAPP‐A MoM were significantly lower than in the non‐CVS group. The gestational age at CVS was significantly higher than the gestational age at first‐trimester scan in the group which did not undergo CVS. Compared with the non‐CVS group, more women in the CVS group were parous, were of black, South Asian or East Asian ethnicity, had chronic hypertension, had conceived by assisted reproductive techniques and had abnormal flow in the fetal ductus venosus. The only parameters that were not significantly different between the two groups were the frequency of pre‐existing diabetes mellitus and of smoking.

**Table 1:** Maternal and pregnancy characteristics of study population of singleton pregnancies assessed at any participating center that underwent chorionic villus sampling (CVS group) and those assessed at Hospital Clínico Universitario Virgen de la Arrixaca in Murcia, Spain, who did not undergo CVS (non‐CVS group)

Variable	Non‐CVS group* (*n* = 22 250)	CVS group (*n* = 3613)	*P* †	Standardized difference (%)
Maternal age (years)	32.5 (28.4–35.8)	35.2 (31.4–38.3)	< 0.0001	49.0
Maternal weight (kg)	64.0 (57.3–73.0)	63.5 (57.0–72.0)	0.0014	–5.4
Maternal height (cm)	163 (160–168)	163 (159–167)	0.0281	–3.4
Ethnicity				6.0
White	21 937 (98.6)	3526 (97.6)	< 0.0001	
Black	221 (1.0)	52 (1.4)	0.0190	
South Asian	21 (0.1)	13 (0.4)	0.0001	
East Asian	71 (0.3)	22 (0.6)	0.0108	
Method of conception			0.0048	4.9
Natural	21 258 (95.5)	3413 (94.5)		
Assisted	992 (4.5)	200 (5.5)		
Parity				18.0
Nulliparous	10 246 (46.0)	1345 (37.2)	< 0.0001	
Parous	12 004 (54.0)	2268 (62.8)	< 0.0001	
Cigarette smoker	3137 (14.1)	467 (12.9)	0.0625	3.4
Medical history				
Diabetes mellitus	223 (1.0)	40 (1.1)	0.4240	1.7
Not known	1846 (8.3)	469 (13.0)	< 0.0001	
Chronic hypertension	157 (0.7)	46 (1.3)	< 0.0001	7.4
Not known	66 (0.3)	510 (14.1)	< 0.0001	
GA (weeks)	12.6 (12.2–13.1)	13.0 (12.5–13.5)	< 0.0001	50.4
Delta NT (mm)	0.16 (–0.06 to 0.40)	0.32 (–0.01 to 0.85)	< 0.0001	43.4
Ductus venosus flow				
Abnormal	1059 (4.8)	384 (10.6)	< 0.0001	26.6
Not known	907 (4.1)	511 (14.1)	< 0.0001	
Free β‐hCG MoM	1.05 (0.69–1.63)	1.29 (0.77–2.12)	< 0.0001	28.9
PAPP‐A MoM	0.94 (0.67–1.34)	0.52 (0.32–0.86)	< 0.0001	–69.1
Miscarriage	207 (0.9)	77 (2.1)	< 0.0001	

Data are given as median (interquartile range) or *n* (%).

* Subset of women included in propensity score regression analysis was obtained from this group.

† Comparisons between outcome groups were by χ^2^ test for categorical variables and Mann–Whitney *U*‐test for continuous variables.

β‐hCG, β‐human chorionic gonadotropin; GA, gestational age at CVS or, if this was not carried out, at first‐trimester scan; MoM, multiples of the median; NT, nuchal translucency; PAPP‐A, pregnancy‐associated plasma protein‐A.

The incidence of miscarriage in the CVS group was 2.1% (77/3613), significantly higher than that in the non‐CVS group (0.9% (207/22 250)) (*P* < 0.0001).

### Procedure‐related risk of miscarriage

We calculated the PS for each case in the study population based on their probability of undergoing CVS. Multivariable logistic regression analysis showed that significant predictors associated with having CVS were increasing maternal age, decreasing maternal weight, assisted conception, chronic hypertension, increasing gestational age, high fetal NT thickness, abnormal flow in the ductus venosus, high free β‐hCG and low PAPP‐A (Table S1).

**Figure 2:**
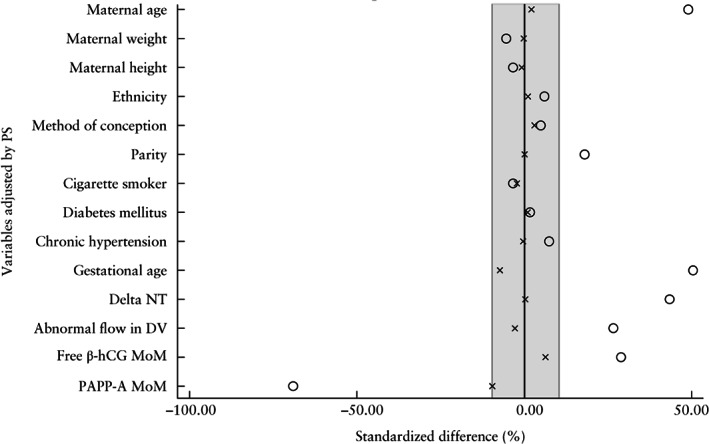
Standardized difference in maternal and pregnancy characteristics between cases that underwent and those that did not undergo chorionic villus sampling, before (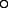) and after (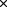) propensity score (PS) matching. Gray shaded area denotes 10% standardized difference between covariates. β‐hCG, β‐human chorionic gonadotropin; DV, ductus venosus; MoM, multiples of the median; NT, nuchal translucency; PAPP‐A, pregnancy‐associated plasma protein‐A.

The PS algorithm matched 2122 of the CVS cases with 2122 non‐CVS pregnancies, largely reducing the initial imbalance between the two groups, with the between‐group standardized differences for all variables being lower than the recommended 10% limit (Figure 2, Tables 1 and 2). There were 40 (1.9%) miscarriages in the CVS group and 55 (2.6%) in the matched non‐CVS group. The PS analysis did not indicate any significant association between CVS and miscarriage (OR, 0.72 (95% CI, 0.48–1.10); *P* = 0.146). We hypothesized that the most likely explanation for this paradoxical effect of CVS ‘reducing’ the risk of miscarriage was that many of the cases that would have resulted in spontaneous miscarriage if the pregnancy had continued underwent elective termination following an abnormal genetic diagnosis. If this is true, this ‘protective’ effect of CVS should be higher in cases at high risk of having a genetic anomaly and lower in low‐risk cases.

**Table 2:** Maternal and pregnancy characteristics of chorionic villus sampling (CVS) group and non‐CVS group, matched by propensity score

Variable	Non‐CVS group (*n* = 2122)	CVS group (*n* = 2122)	*P* *	Standardized difference (%)
Maternal age (years)	34.8 (31.5–37.7)	34.7 (31.1–37.9)	0.5789	2.1
Maternal weight (kg)	63.0 (57.0–71.5)	63.0 (56.6–71.2)	0.9949	–0.2
Maternal height (cm)	163 (159–167)	163 (159–167)	0.9582	–0.8
Ethnicity			0.8592	1.1
White	2107 (99.3)	2105 (99.2)		
Non‐white	15 (0.7)	17 (0.8)		
Method of conception			0.3681	3.0
Natural	2019 (95.1)	2005 (94.5)		
Assisted	103 (4.9)	117 (5.5)		
Parity			1.000	0.1
Nulliparous	853 (40.2)	854 (40.2)		
Parous	1269 (59.8)	1268 (59.8)		
Cigarette smoker	288 (13.6)	272 (12.8)	0.4963	–2.2
Medical history				
Diabetes mellitus	20 (0.9)	23 (1.1)	0.8669	1.0
Chronic hypertension	27 (1.3)	26 (1.2)	1.000	–0.4
Gestational age (weeks)	13.0 (12.5–13.4)	12.9 (12.4–13.4)	0.0414	–7.3
Delta NT (mm)	0.33 (0.08 to 0.65)	0.26 (–0.02 to 0.65)	< 0.0001	0.3
Abnormal flow in ductus venosus	251 (11.8)	232 (10.9)	0.3843	–2.8
Free β‐hCG MoM	1.19 (0.74–1.91)	1.22 (0.75–1.96)	0.5273	6.3
PAPP‐A MoM	0.66 (0.48–0.90)	0.52 (0.32–0.87)	< 0.0001	–9.9
Miscarriage	55 (2.6)	40 (1.9)	0.1463	

Data are given as median (interquartile range) or *n* (%).

Covariates used to identify matched women who did not undergo CVS were maternal age, weight, height and ethnicity, method of conception, parity, smoking status, chronic hypertension, gestational age, nuchal translucency (NT), free β‐human chorionic gonadotropin (β‐hCG) and pregnancy‐associated plasma protein‐A (PAPP‐A).

* Comparisons between outcome groups were by χ^2^ test for categorical variables and Mann–Whitney *U*‐test for continuous variables.

**Figure 3:**
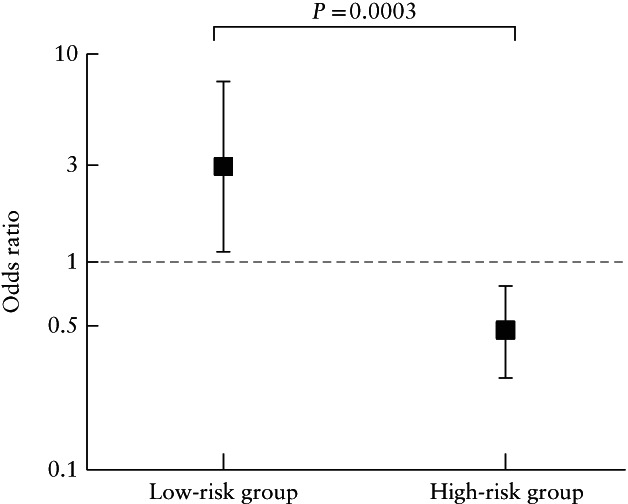
Odds ratio (95% CI) for miscarriage following chorionic villus sampling (CVS) in singleton pregnancies with high and those with low risk of having CVS (following high‐ or low‐risk result, respectively, on screening for aneuploidy), matched according to propensity score.

Therefore, we aimed to investigate whether the effect of CVS was the same in women at higher risk and those at lower risk for aneuploidy. To do so, we assessed the possible interaction between the risk of aneuploidy and CVS. Since the risk factors associated with having CVS are also associated with an increased risk for aneuploidy, we divided our 4244 matched cases into two equal groups by the median of the PS. The median PS was 0.402 (IQR, 0.331–0.490) in the high‐risk subgroup (*n* = 2122) and 0.131 (IQR, 0.057–0.197) in the low‐risk subgroup (*n* = 2122). In the high‐risk subgroup, 1062 cases underwent CVS, of which 23 (2.2%) had a miscarriage, and of the 1060 cases that did not undergo CVS, 49 (4.6%) had a miscarriage (OR, 0.47 (95% CI, 0.28–0.76)). In contrast, in the low‐risk subgroup, 17 (1.6%) miscarriages occurred in the CVS group (*n* = 1060) compared with six (0.6%) miscarriages in the non‐CVS group (*n* = 1062) (OR, 2.87 (95% CI, 1.13–7.30)). Both effects were statistically significantly different (*P* for the interaction = 0.0003) (Figure 3). These findings suggest that the effect of CVS on the risk of miscarriage is different when the risk of aneuploidy is high compared to when it is low. Thus, using the PS as a proxy for the risk for aneuploidy, for a patient with a 10% probability of aneuploidy based on her pregnancy characteristics, the risk of miscarriage after CVS is still very high but halved to about 5%, suggesting that in such cases CVS is highly ‘protective’ of miscarriage. However, in a patient with a low probability of aneuploidy the risk of miscarriage increases after CVS. For example, if the risk of aneuploidy is 1 in 1000 (0.1%), the risk of miscarriage after CVS will increase to 0.3% (0.2 percentage points higher) or, in other words, one additional miscarriage would occur for every 500 CVS procedures. Further analysis on this interaction is provided in Appendix S1.

## DISCUSSION

### Main findings

This study found that, first, following a first‐trimester scan demonstrating a structurally normal fetus, the risk of subsequent miscarriage in the general population is about 1%; second, in women undergoing CVS the risk of miscarriage is about 1% higher than that in women who do not have CVS, although this excess risk is not attributed solely to the invasive procedure but, to some extent, to the demographic and pregnancy characteristics of the patient; and third, the actual procedure‐related risk of CVS may only become apparent in patients at low risk for aneuploidy and, in these cases, the risk of miscarriage after CVS increases by about three times.

We have shown that, although in women at high risk for aneuploidy CVS appears to be ‘protective’ against miscarriage, the most likely explanation for this observation is that CVS leads to the diagnosis of major aneuploidies followed by elective termination of pregnancy in cases that would otherwise have resulted in spontaneous miscarriage. In the CVS group we excluded 22.2% (1135/5112) of cases because of termination of pregnancy or fetal defects, compared with only 4.2% (1070/25 519) in the non‐CVS group (Figure 1). If these cases had been included in the analysis and the pregnancy had continued, many would have resulted in miscarriage and then the rate of miscarriage in the CVS group would have been considerably higher than that in the non‐CVS group. To try to avoid this selection bias, we studied separately the effect of CVS in cases with a low and in those with a higher probability of having a CVS. Contrary to what happens in high‐risk cases, in women at low risk of aneuploidy, the procedure significantly increases the risk of miscarriage by about three times.

### Comparison with findings of previous studies

Our results offer an explanation for the contradictory findings of previous studies that showed that CVS did not significantly alter the risk of miscarriage^13^, ^14^, ^15^, ^16^, and a meta‐analysis that reported a non‐significant ‘protective’ effect of CVS against miscarriage^19^.

A large study examined 31 460 pregnancies undergoing first‐trimester combined screening for aneuploidy without CVS and identified risk factors for miscarriage^13^. This model was then applied to 2396 pregnancies that had CVS and found that the estimated number of miscarriages was 45 (95% CI, 32–58), which was similar to the observed number of 44^13^. Two subsequent studies followed a similar methodology and did not find significant differences between the groups^14^, ^15^.

A large national registry‐based study assessing 147 987 singleton pregnancies that underwent first‐trimester combined screening for aneuploidy, including 5072 that had CVS, reported that the average effect of CVS on the risk of miscarriage was a change of –0.21% (95% CI, –0.58 to 0.15%)^16^. In that study, the CVS‐related risk of miscarriage was assessed by a dynamic PS stratification approach^16^. The advantage of this approach is that it allows use of the whole sample, but the major disadvantage is that the higher the number of cases per stratum the greater is the difference in baseline characteristics of the patients, even within the same stratum. In our matching approach, we used a 1:1 ratio and a small difference in PS between matched cases (caliper of 0.1) to ensure that the CVS and non‐CVS groups had a very similar risk profile.

A recent RCT randomized women at high risk of aneuploidy into cell‐free DNA testing (*n* = 1015) or invasive testing (amniocentesis or CVS (*n* = 982)), and found no significant differences in the risk of miscarriage between the two groups (0.8% *vs* 0.8%, for a risk difference of –0.03% (one‐sided 95% CI, −0.68% to ∞); *P* = 0.47)^27^.

### Clinical implications

In cases in which there is a clear indication for performing prenatal genetic testing, clinicians can reassure women that their risk of miscarriage mainly depends on the results of the genetic diagnosis and the conditions that led to it more than the procedure itself. However, in the absence of any major fetal defect or other additional risk factors for chromosomal abnormality, clinicians should report an individualized procedure‐related risk based on the clinical characteristics of the patient.

### Strengths and limitations

The main limitations of this study are related to its observational and retrospective nature, and the consequent heterogeneity between the two study groups (Figure 2). Although we tried to mitigate these differences, we were able to balance only those maternal and pregnancy characteristics that had been recorded, therefore we cannot disregard the possibility of residual confounding. Additionally, we could not assess the influence of technical factors or the experience of the clinician who performed the procedure on the risk of miscarriage, since these data are not routinely recorded in any of the participating centers; however, these are already well‐known risk factors^28^ ^,^ ^29^. Fetal karyotype was not available in most cases that had spontaneous miscarriage, therefore our assumption of increased rate of aneuploidies among these cases remains hypothetical. We chose to exclude pregnancies with aneuploidy or fetal defects from the analysis to avoid overestimation of the risk of miscarriage in the CVS group, since these cases are highly likely to result in miscarriage. However, this exclusion inevitably produced the opposite effect, as shown by our findings, resulting in underestimation of the procedure‐related risk of miscarriage owing to lack of knowledge of the karyotype in most of the miscarriages in the non‐CVS group, while the CVS sample was ‘clean’ of aneuploidies.

The main strength of our study relates to the large sample of both CVS and non‐CVS cases, which were selected after matching women of both groups but with identical PS for having a CVS. Since the matching was indirectly based on known risk factors for aneuploidy, we were able to perform subgroup analysis to demonstrate the interaction between the risk of aneuploidy and CVS by comparing patients with a very similar risk profile.

All invasive procedures were performed using the same technique and by fetal medicine experts or their trainees at the end of such training. This represents both an advantage, because it reduces variability between operators, and a disadvantage, since the results might not be valid for different approaches and levels of expertise.

### Conclusions

The risk of miscarriage in pregnancies that undergo CVS is about 1% higher than that in pregnancies that do not have CVS, although this excess risk is not attributed entirely to the invasive procedure but to some extent to the demographic and pregnancy characteristics of the patient. After adjusting for these risk factors and confining the analysis to low‐risk pregnancies, CVS seems to increase the risk of miscarriage by about three times above the patient's background risk. Although this is a substantial increase in relative terms, in pregnancies without risk factors, the risk of miscarriage after CVS will remain low and similar to, or slightly higher than, that in the general population.

## Supporting information


**Table S1** Additional statistics

**Appendix S1** Propensity score analysis according to background risk for miscarriage
