# Supplementary material for: New approach for estimating risk of miscarriage after chorionic villus sampling
Source: Ultrasound Obstet Gynecol. 2020 Oct 17;56(5):656–63. doi: 10.1002/uog.22041 (PMC7984173; doi:10.1002/uog.22041)
Supplement: Supplementary file 1 — Table S1 Additional statistics [file UOG-56-656-s002.docx]

**Table S1**. Logistic regression model for prediction of having chorionic villus sampling, used for calculation of propensity score

| **Variable** | **Coefficient** | **SE** | **95% CI** | ***P* value** |
| --- | --- | --- | --- | --- |
| Intercept | -14.5878 | 0.849 | (-16.257, -12.929) | <0.0001 |
| Maternal age (per year) | 0.0932 | 0.005 | (0.083, 0.103) | <0.0001 |
| Maternal weight (per kg) | -0.0152 | 0.002 | (-0.019, -0.011) | <0.0001 |
| Maternal height (per cm) | 0.0045 | 0.004 | (-0.004, 0.013) | 0.2841 |
| Non-White racial origin | -0.2762 | 0.259 | (-0.809, 0.210) | 0.2859 |
| Assisted conception | -0.3344 | 0.115 | (-0.564, -0.111) | 0.0038 |
| Parous | -0.0045 | 0.052 | (-0.107, 0.098) | 0.9309 |
| Cigarette smoker | -0.0094 | 0.073 | (- 0.154,0.132) | 0.8977 |
| Chronic hypertension | 0.4852 | 0.223 | (0.036, 0.914) | 0.0299 |
| Gestational age (per day) | 0.1182 | 0.006 | (0.107, 0.130) | <0.0001 |
| Delta nuchal translucency (per mm) | 1.3080 | 0.047 | (1.217, 1.400) | <0.0001 |
| Abnormal flow in the ductus venosus | 1.1251 | 0.083 | (0.962, 1.287) | <0.0001 |
| Free β-hCG (per MoM) | 0.4346 | 0.023 | (0.390, 0.480) | <0.0001 |
| PAPP-A (per MoM) | -2.2919 | 0.069 | (-2.429, -2.158) | <0.0001 |

hCG = human chorionic gonadotropin; PAPP-A = pregnancy associated plasma protein-A; SE = Standard error; CI = confidence interval.
