# Supplementary material for: New approach for estimating risk of miscarriage after chorionic villus sampling
Source: Ultrasound Obstet Gynecol. 2020 Oct 17;56(5):656–63. doi: 10.1002/uog.22041 (PMC7984173; doi:10.1002/uog.22041)
Supplement: Supplementary file 2 — Appendix S1 Propensity score analysis according to background risk for miscarriage [file UOG-56-656-s001.docx]

**APPENDIX S1: Propensity score analysis according to background risk for miscarriage**

The logistic regression model for propensity score (PS) calculation in the study population is provided in table S1.

Once we demonstrated the interaction between PS and CVS in our subset of matched cases, we wanted to investigate further the change in the posterior risk after CVS for different background risk. By logistic regression analysis, we fitted a model with miscarriage as the dependent binary variable and CVS, PS and their interaction as independent variables in all patients with available PS (CVS, n=2,539; non-CVS, n=17,189). The estimated OR were 2.46 (95% CI 1.35 to 4.30) for CVS, 140.6 (95% CI 70.8 to 273.5) for PS and 0.03 (95% CI 0.01 to 0.11) for the interaction between CVS and PS. Therefore, the risk of miscarriage after CVS varies according to the PS; there is an inverse relation between the CVS-related risk of miscarriage and PS which is given by the equation: ${OR}_{CVS}=2.459 \times{0.0322}^{PS}$. This model allows estimation of the CVS-related risk for each probability of receiving a CVS, treated both as continuous variables.

**Table S1**. Logistic regression model for prediction of having a chorionic villus sampling used for calculation of the propensity score.

| **Variable** | **Coefficient** | **SE** | **95% CI** | ***P* value** |
| --- | --- | --- | --- | --- |
| Intercept | -14.5878 | 0.849 | (-16.257, -12.929) | <0.0001 |
| Maternal age (per year) | 0.0932 | 0.005 | (0.083, 0.103) | <0.0001 |
| Maternal weight (per kg) | -0.0152 | 0.002 | (-0.019, -0.011) | <0.0001 |
| Maternal height (per cm) | 0.0045 | 0.004 | (-0.004, 0.013) | 0.2841 |
| Non-White racial origin | -0.2762 | 0.259 | (-0.809, 0.210) | 0.2859 |
| Assisted conception | -0.3344 | 0.115 | (-0.564, -0.111) | 0.0038 |
| Parous | -0.0045 | 0.052 | (-0.107, 0.098) | 0.9309 |
| Cigarette smoker | -0.0094 | 0.073 | (- 0.154,0.132) | 0.8977 |
| Chronic hypertension | 0.4852 | 0.223 | (0.036, 0.914) | 0.0299 |
| Gestational age (per day) | 0.1182 | 0.006 | (0.107, 0.130) | <0.0001 |
| Delta nuchal translucency (per mm) | 1.3080 | 0.047 | (1.217, 1.400) | <0.0001 |
| Abnormal flow in the ductus venosus | 1.1251 | 0.083 | (0.962, 1.287) | <0.0001 |
| Free β-hCG (per MoM) | 0.4346 | 0.023 | (0.390, 0.480) | <0.0001 |
| PAPP-A (per MoM) | -2.2919 | 0.069 | (-2.429, -2.158) | <0.0001 |

hCG = human chorionic gonadotropin; PAPP-A = pregnancy associated plasma protein-A; SE = Standard error; CI = confidence interval.
